# Synthesis and Characterization of Cobalt NCN Pincer Complexes

**DOI:** 10.1002/ejic.202100643

**Published:** 2021-10-11

**Authors:** Jan Pecak, Wolfgang Eder, Gerald Tomsu, Berthold Stöger, Marc Pignitter, Karl Kirchner

**Affiliations:** ^1^ Institute of Applied Synthetic Chemistry Vienna University of Technology Getreidemarkt 9 A-1060 Wien Austria; ^2^ X-Ray Centre Vienna University of Technology Getreidemarkt 9 1060 Wien Austria; ^3^ Department of Physiological Chemistry, Faculty of Chemistry University of Vienna Althanstraße 14 1090 Wien Austria

**Keywords:** Cobalt, DFT studies, EPR studies, Nitrosyl ligand, Pincer complexes

## Abstract

A series of cobalt complexes, stabilized by a monoanionic tridentate NCN pincer ligand, was synthetized and characterized. Preparation of the paramagnetic 15 VE complex [Co(NCN^CH2^−Et)Br] (**1**) was accomplished by transmetalation of Li[2,6‐(Et_2_NCH_2_)_2_C_6_H_3_] with CoBr_2_ in THF. Treatment of this air‐sensitive compound with NO gas resulted in the formation of the diamagnetic Co(III) species [Co(NCN^CH2^−Et)(NO)Br] (**2**) as confirmed by X‐ray diffraction. This complex features a strongly bent NO ligand (Co−N−O∠135.0°). The ν_NO_ is observed at 1609 cm^−1^ which is typical for a bent metal‐N−O arrangement. Coordinatively unsaturated **1** could further be treated with pyridine, isocyanides, phosphines and CO to form five‐coordinate 17 VE complexes. Oxidation of **1** with CuBr_2_ led to the formation of the Co(III) complex [Co(NCN^CH2^−Et)Br_2_]. Treatment of [Co(NCN^CH2^−Et)Br_2_] with TlBF_4_ as halide scavenger in acetonitrile led to the formation of the cationic octahedral complex [Co(NCN^CH2^−Et)(MeCN)_3_](BF_4_)_2_. A combination of X‐ray crystallography, IR‐, NMR‐ and EPR‐spectroscopy as well as DFT/CAS‐SCF calculations were used to characterize all compounds.

## Graphical abstract

The preparation of several Co(II) and Co(III) NCN pincer complexes is described. A combination of X‐ray diffraction, EPR‐, IR‐ and NMR spectroscopy together with computational methods was used to characterize and study the properties of all products.

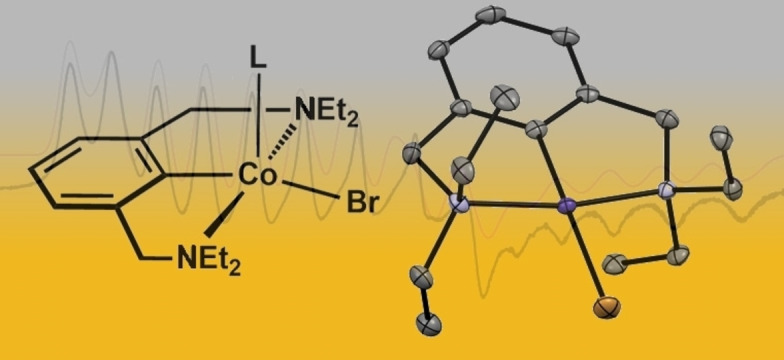

## Introduction

**Scheme 1:**
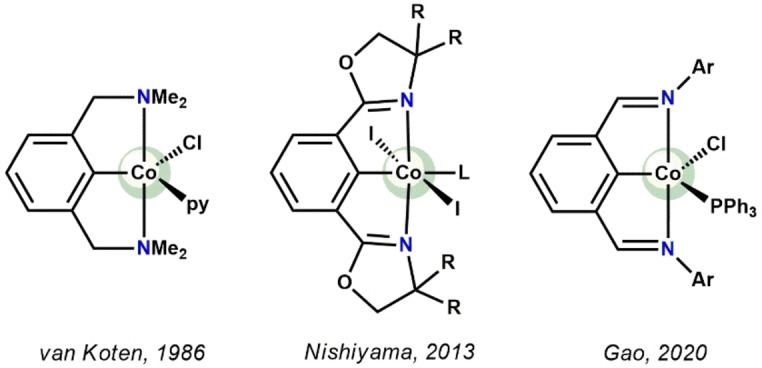
Literature known NCN cobalt pincer complexes.

Despite the wide prominence of pincer complexes^[1]^ with phosphine donors and their diversity amongst d‐block elements, the chemistry of NCN ([2,6‐(R_2_NCH_2_)_2_C_6_H_3_]^−^, R=alkyl) pincer transition metal complexes is rich but largely limited to Ni, Pd, and Pt. Most notably by van Koten and coworkers, numerous Pd and Pt complexes have been prepared for applications in catalysis, sensor systems or even as building blocks for biomolecular and peptide chemistry.^[2]^ Ever since the first PCP systems were reported, pincer ligands evolved to be extremely valuable scaffolds for stabilization of transition metal fragments in various configurations and oxidation states. The major difference affecting coordination chemistry of the NCN ligand, is the N‐atom being significantly smaller than the corresponding P‐atom in PNP or PCP ligands and the aliphatic NR_2_ group acting exclusively as a σ‐donor. Moreover, NCN ligands are coordinated in typically in planar tridentate *mer*‐fashion, but in some cases also a *fac* geometry was observed.^[2g]^ It was also shown that a direct regio‐selective C_arene_−H activation of the *ipso* position is unfavorable when strongly coordinating groups (σ and π) are missing, a problem that is well known for simple metal salts and for thermodynamic reasons. It needs to be mentioned that aside transition metal coordination chemistry, NCN^CH2^−R (R=alkyl) systems were also reported to successfully stabilize main group elements such as Ge, Sn, and Te.^[3]^ As far as cobalt is concerned, only the κ^3^‐NCN bis(amino)aryl complex [Co(2,6‐(Me_2_NCH_2_)_2_C_6_H_3_)X(L)] (X=Cl, Br, L=py, PPh_3_) was reported by van Koten in 1986 and studied by EPR and UV‐VIS‐NIR spectroscopy.^[4]^ Aside bis(amino)aryl ligands, bis(imino)aryl and bis(oxazolinyl) ligands constitute important representatives of NCN pincer systems and a few cobalt complexes are known thereof (Scheme 1).[^5^, ^6^]

In this contribution we report on the synthesis and characterization of several new cobalt NCN pincer complexes. X‐Ray structures, EPR‐spectra, and DFT/CAS‐SCF calculations are presented.

## Results and Discussion

**Scheme 2:**
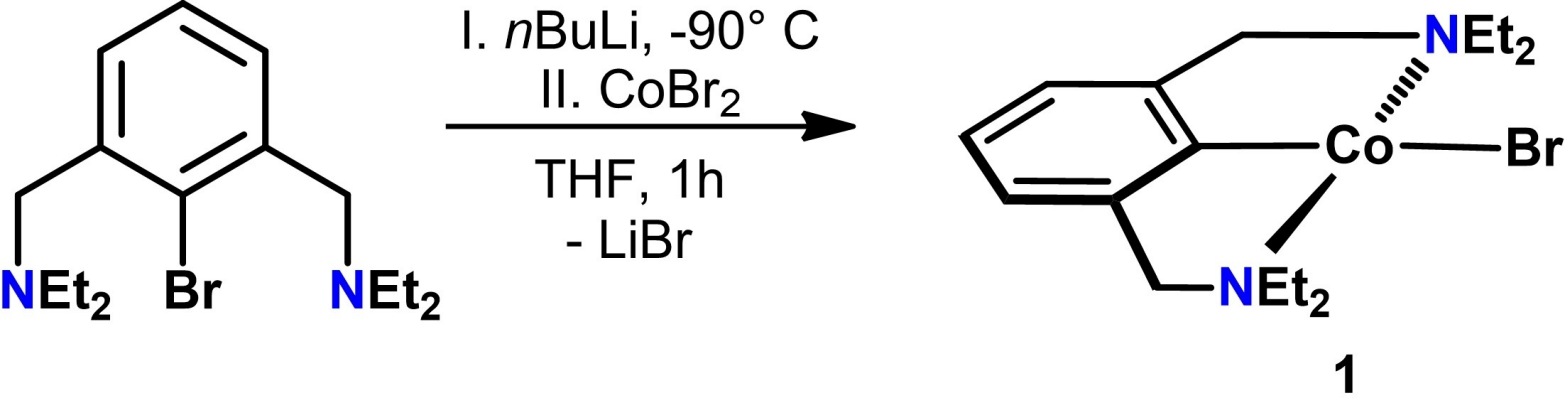
Synthesis of complex **1** *via* transmetalation.

**Figure 1:**
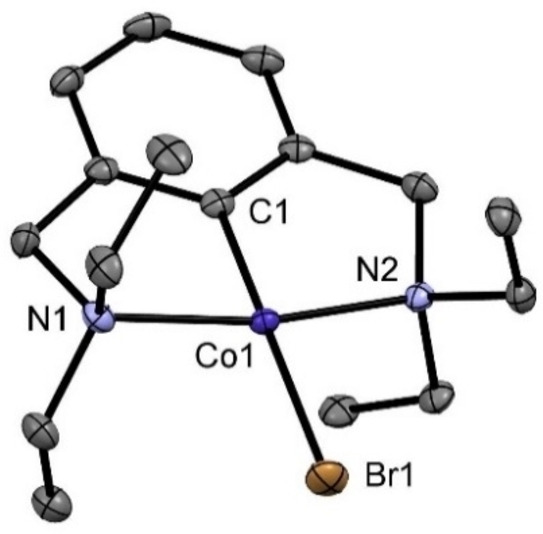
Structural view of [Co(NCN^CH2^−Et)Br] (**1**) showing 50 % displacement ellipsoids (H atoms omitted for clarity). Selected bond lengths [Å] and angles [°]: C1−Co1 1.850(3), Co1−Br1 2.438(3), Co1−N1 2.033(3), Co1−N2 2.044(3), N1−Co1−N2 167.76(9), C1−Co1−Br1 179.35(8), C1−Co1−N1 83.9(1).

In an attempt to reappraise van Koten's seminal work, we used a direct lithiation protocol starting from the free ligand N(C−Br)N^CH2^−Et that itself was prepared by reacting bis(benzylic bromide) with diethyl amine at room temperature.^[7]^ Treatment of the lithium species with stoichiometric amount of CoBr_2_ suspended in THF at low temperature resulted in a color change to dark violet. After careful workup, the highly air sensitive complex [Co(NCN^CH2^−Et)Br] (**1**) was obtained in 67 % isolated yield (Scheme 2). The measurement of the solution magnetic properties (Evans method, benzene) revealed an effective magnetic moment of 2.3(1) μ_B_. This value is in agreement with other reported Co(II) PCP pincer complexes suggesting a d^7^ low spin system. In order to unequivocally establish the ligand arrangement and geometry, single crystals were grown from a saturated pentane solution kept at −20 °C. A view of the molecular structure is depicted in Figure 1 with selected metrical parameters reported in captions. The complex adopts a square planar conformation with almost C_2_ molecular symmetry.

The NCN ligand is coordinated to the metal center in a tridentate meridional fashion. The C1−Co1−Br1 angle is essentially linearly being 179.35(8)° and the N1−Co1−N2 angle is 167.76(9)°. The Co1−C1 distance of 1.850(3) Å is significantly shorter than in corresponding PCP^CH2^ and PCP^O^ (*cf* 1.955 and 1.914 Å) complexes.^[8]^

**Figure 2:**
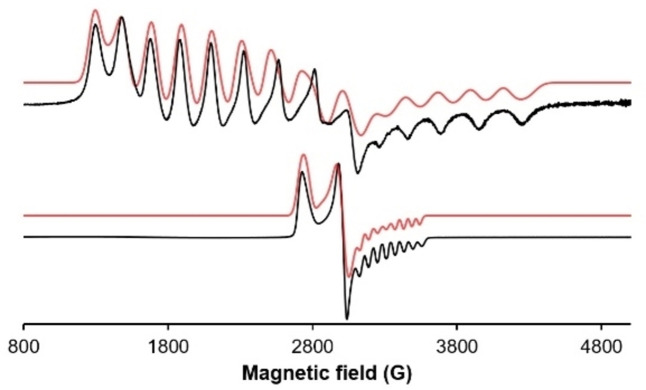
EPR spectra of complexes **1** (top) and **3 a** (bottom) in toluene glass at 100 K microwave frequency of 9.43 GHz and microwave power 15.9 mW. Associated simulations are depicted in red color.

The EPR spectrum of **1** recorded at 100 K shows a strongly broadened rhombic signal with g_1_=3.387, g_2_=2.958 and g_3_=1.953 (g_iso_=2.766) as shown in Figure 2 (top). The hyperfine couplings to ^59^Co (I=7/2) are clearly resolved with coupling constants of A_1_=200 G, A_2_=226 G and A_3_=230 G whereas couplings to the NCN scaffold could not be observed or simulated. The high anisotropy and large values for A(Co) are likely caused by strong spin‐orbit coupling and a nearly degenerate set of d‐orbitals.^[9]^ The present data are consistent with a Co(II) S=1/2 system with the unpaired electron highly localized on the metal center rather than on the ligand.

**Figure 3:**
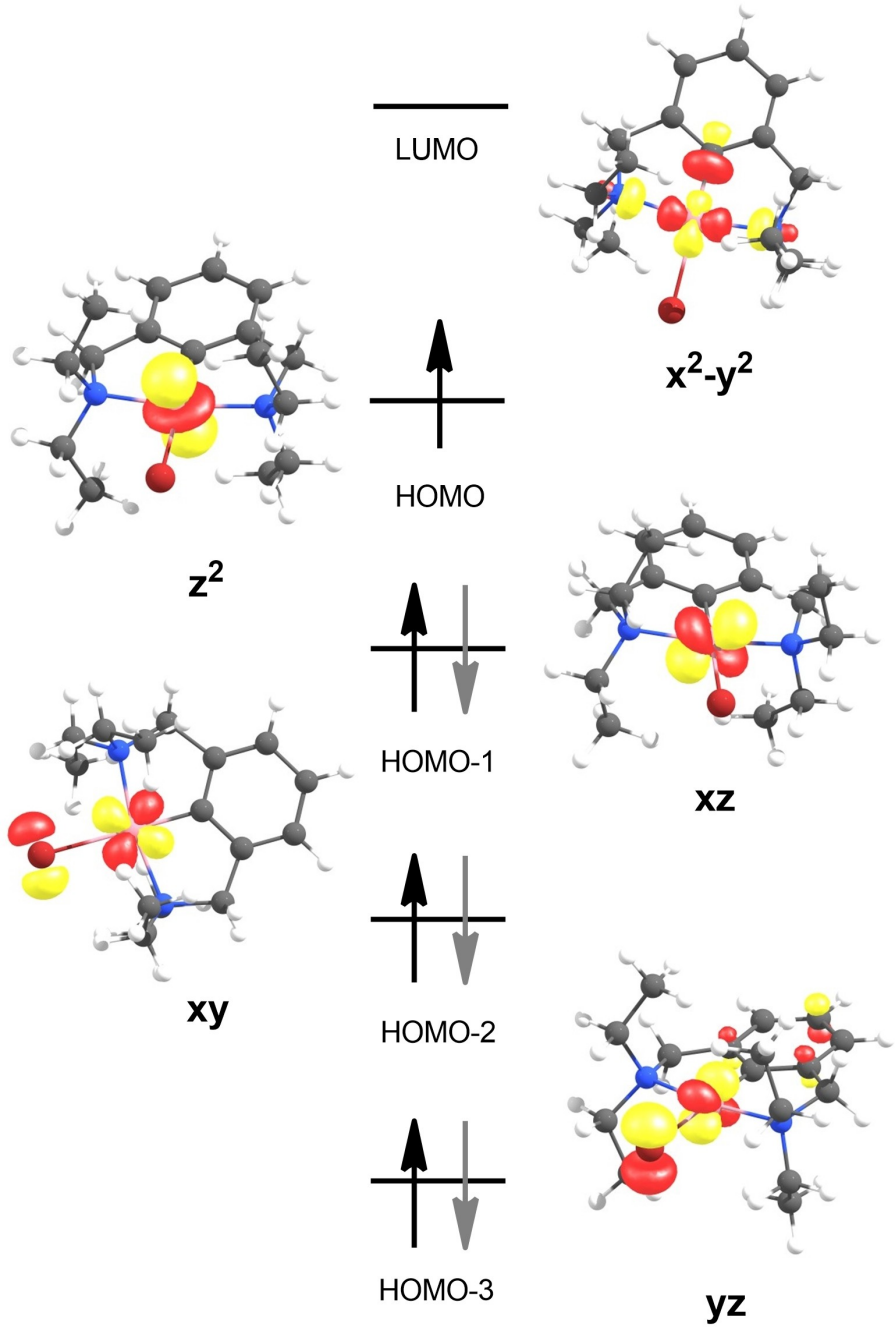
BP86/def2‐TZVP computed frontier orbitals (d‐splitting) for [Co(NCN^CH2^−Et)Br] (**1**).

The electronic structure of [Co^II^(NCN^CH2^−Et)Br] (**1**) was further evaluated by means of computational chemistry. A DFT (BP86/def2‐TZVP) optimized structure of the low spin species on a full model agrees favorably with the metrical parameters of the experimentally determined molecular structure of **1**. DFT calculations reveal that a high spin species (S=3/2) is 15.6 kcal/mol less stable than the doublet species and therefore not observed. Figure 3 shows the qualitative d‐splitting obtained from the alpha MO set with the dz^2^ orbital being the SOMO and the dx^2^–y^2^ being the LUMO consistent with a d^7^ configuration. To confirm these results and better understand the EPR experiment a complete active space self‐consistent field (CAS‐SCF) calculation was performed with additional NEVPT2 correction of the wavefunction (see Supporting Information).^[10]^ The CAS(7,5) calculation supports the DFT results and gives a ground state configuration (96 %) of (d_xy_)^2^(d_xz_)^2^(d_yz_)^2^(d_z2_)^1^(d_x2–y2_)^0^. Within this methodology the g‐values were computed to g_x_=1.91, g_y_=2.81 and g_z_=3.13 (A>150 G) and thus agree satisfactorily well with the experiment.

**Scheme 3:**
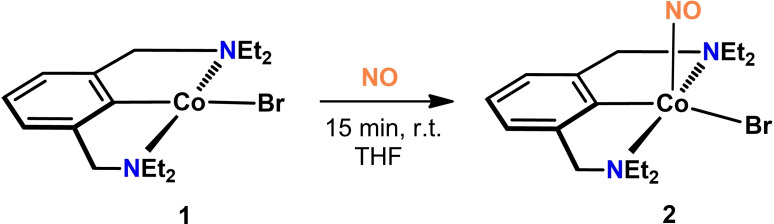
Synthesis of the nitrosyl complex [Co(NCN^CH2^−Et)(NO)Br] (**2**).

In analogy to our previous studies on {CoNO}^8^ complexes, [Co(NCN^CH2^−Et)Br] reacts with NO gas to form the closed‐shell diamagnetic complex [Co(NCN^CH2^−Et)(NO)Br] (**2**) (Scheme 3).^[8]^ In the infrared spectrum **2** exhibits one strong band at 1609 cm^−1^ which is comparable to related cobalt PCP nitrosyl systems and characteristic for a bent coordination mode of the Co−NO moiety. This value indicates the formal presence of an NO^−^ anion and therefore suggests a Co(III) oxidation state. In the ^1^H NMR spectrum the aliphatic protons are giving rise to two separate signals each for C*H*_2_, C*H*_3_ and C*H*_2_NEt_2_, respectively as proved by an ^1^H,^13^C−HSQC NMR experiment. The signals of the C*H*_2_NEt_2_ (linker) protons give rise to resonances at 3.26 and 2.76 ppm (c.f. 3.72 in the free ligand, C_6_D_6_) and show coupling to each other as evidenced by ^1^H,^1^H‐COSY NMR. The inspection of the ^13^C{^1^H} NMR suggests the presence of a second minor species that could be an isomer not found in the solid state. Earlier studies on Ni and Pt NCN pincer complexes already demonstrated the complexity of NMR spectra and the possibility of stereo isomerism.^[11]^

**Figure 4:**
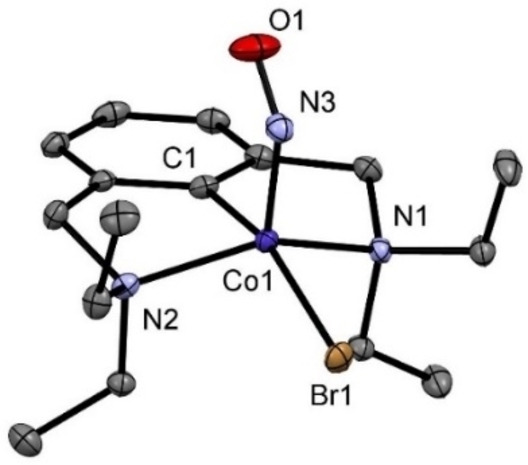
Structural view of [Co(NCN^CH2^−Et)(NO)Br] (**2**) showing 50 % displacement ellipsoids (H atoms omitted for clarity). Selected bond lengths [Å] and angles [°]: Co1−C1 1.883(2), Co1−Br1 2.4915(4), Co1−N1 2.0753(18), Co1−N2 2.0641(19), Co1−N3 1.7400(17), N3−O1 1.178(2), C1−Co1−Br1 162.84(6), N1−Co1−N2 143.71(7), C1−Co1−N3 93.72(9), Co1−N3−O1 135.0(2).

The solid‐state structure of **2** was determined by single crystal X‐ray diffraction. Suitable crystals were grown from a saturated pentane solution kept at −20 °C. A view of the molecular structure is depicted in Figure 4 with selected bond distances and angles reported in captions. The complex adopts a distorted square pyramidal geometry (τ_5_=0.31)^[12]^ with the NCN ligand coordinated in a tridentate fashion to the metal center and almost C_S_ point group symmetry. The N−O group is occupying the apical position and is strongly bent towards the aromatic scaffold. The N−O bond distance is 1.178(2) Å and the Co−N−O angle is 135.0(2)° both in accordance with earlier reported {CoNO}^8^ PCP pincer complexes. All attempts to generate the cationic complex [Co(NCN^CH2^−Et)(NO)]^+^ using halide scavengers such as AgBF_4_ or TlBF_4_ failed and resulted in partial decomposition of the starting material.

**Scheme 4:**
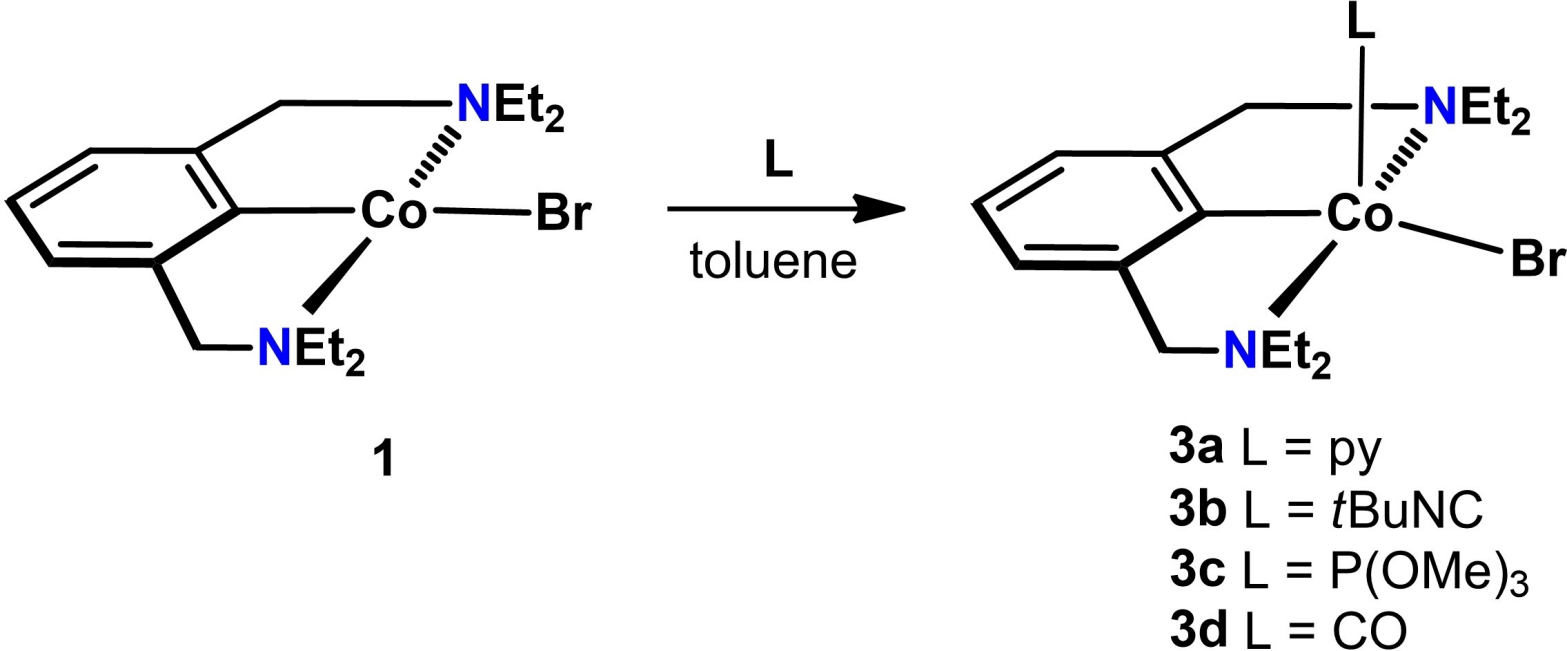
Synthesis of 17 VE complexes **3 a**–**d**.

Due to the sensitivity of the generated substances, *in situ* spectroscopic experiments were performed wherein coordinatively unsaturated complex **1** was reacted with L=pyridine, *t*BuNC, P(OMe)_3_ and CO to form complexes tentatively assigned as [Co(NCN^CH2^−Et)(L)Br] (**3 a**–**d**) (Scheme 4). These 17 VE complexes were not isolated but directly studied by X‐Band EPR spectroscopy in frozen toluene glass and IR spectroscopy in the case of **3 d**. In comparison to complex **1**, complex **3 a** gives rise to a much more compact signal with g_1_=2.017, g_2_=2.228 and g_3_=2.459 (g_iso_=2.235). The hyperfine couplings to ^59^Co are well resolved with A_zz_=58 G and comparable with van Koten's earlier contribution.^[4]^ The spectra for the *t*BuNC and P(OMe)_3_ coordinated species show similarly well resolved signals with g_iso_=2.120 and g_iso_=2.144, respectively (see Supporting Information). The infrared spectrum of **3 d** gives rise to a distinctive band at 1967 cm^−1^ which is in accordance with earlier reported PCP cobalt mono carbonyl complexes.^[13]^ An analogous computational protocol (*vide supra*) was applied to study complex **3 a** and to theoretically justify the strongly differing EPR parameters with respect to **1**. The structure of the putative pyridine adduct was optimized using DFT on a full model and used for a consecutive CAS(7,5)/NEVPT2 calculation (96 % of configuration [22210]). The EPR parameters were calculated to g_x_=2.01, g_y_=2.19 and g_z_=2.60 agreeing with the observed experimental trend.

**Scheme 5:**
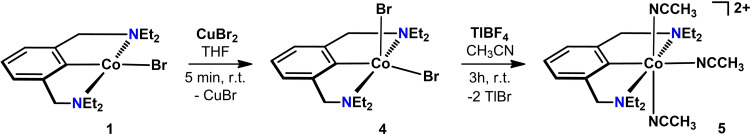
Synthesis of the Co(III) species **4** and **5**.

The oxidation of complex **1** with Cu(II) bromide in THF leads to a color change from violet to green and formation of the paramagnetic five‐coordinate Co(III) species [Co(NCN^CH2^−Et)Br_2_] (**4**) in 88 % isolated yield (Scheme 5). The solution magnetic moment (Evans method, THF) of 4.8(1) μ_B_ is consistent with a d^6^ high spin system, corresponding to four unpaired electrons, and is within the observed range of other five‐coordinate Co(III) complexes known. Compound **4** was then treated with TlBF_4_ and in acetonitrile as solvent to generate the diamagnetic tris acetonitrile complex [Co(NCN^CH2^−*i*Pr)(CH_3_CN)_3_]^2+^ (**5**). Surprisingly, this complex turned out to be very unstable and all attempts to isolate this compound in pure form failed due to decomposition and formation of intractable paramagnetic compounds. This compound was thus merely characterized by ^1^H NMR spectroscopy. This behavior is in sharp contrast to the analogous Co(III) complex [Co(PCP^NMe^−*i*Pr)(CH_3_CN)_3_]^2+^ bearing PCP ligands described previously.^[13]^

## Conclusion

The preparation of several Co(II) and Co(III) NCN pincer complexes is described. A simple transmetalation protocol allowed for the synthesis of the highly air‐sensitive 15 VE complex [Co^II^(NCN^CH2^−Et)Br] (**1**) which provided the starting material for subsequent transformations. The reaction with NO gas yields the diamagnetic {CoNO}^8^ species [Co(NCN^CH2^−Et)(NO)Br] featuring a strongly bent NO ligand (Co−N−O∠135.0°). The ν_NO_ is observed at 1609 cm^−1^ which is typical for a bent metal‐N−O arrangement. Addition of various co‐ligands L=py, *t*BuCN, P(OMe)_3_, and CO to **1** in toluene leads to the formation of very unstable and not isolable five‐coordinate complexes of the type [Co(NCN^CH2^−Et)(L)Br]. Oxidation of **1** with CuBr_2_ results in the formation of the high‐spin complex [Co^III^(NCN^CH2^−Et)Br_2_] that can be transformed into the diamagnetic, but very unstable, tris‐acetonitrile complex [Co^III^(NCN^CH2^−Et)(MeCN)_3_]^2+^. A combination of X‐ray diffraction, EPR‐, IR‐ and NMR spectroscopy together with computational methods was used to characterize and study the properties of all products.

## Experimental section

### General information

All manipulations were performed under an inert atmosphere of Argon by using Schlenk techniques or in an MBraun inert‐gas glovebox. The solvents were purified according to standard procedures. The deuterated solvents were purchased from Aldrich and dried over 4 Å molecular sieves. Nitric oxide (NO 2.5) was purchased from MESSER GmbH (Gumpoldskirchen, Austria). The ligand N(C−Br)N^CH2^−Et was synthesized according to literature and purified *via* distillation.^14 1^H, ^13^C{^1^H}, and COSY NMR spectra were recorded on an AVANCE‐400 spectrometer. ^1^H and ^13^C{^1^H} NMR spectra were referenced internally to residual protio‐solvent and solvent resonances, respectively, and are reported relative to tetramethylsilane (δ=0 ppm). Infrared spectra were recorded in attenuated total reflection (ATR) mode on a PerkinElmer Spectrum Two FT‐IR spectrometer. Elemental analysis was performed on an elementar vario MACRO (Elementar Analysensysteme GmbH, Germany) CHNS analyzer. High‐resolution mass spectra were recorded on an Agilent 6545 QTOF equipped with an Agilent Dual AJS ESI ion source (Agilent Technologies, Santa Clara, USA). Measured accurate mass data for confirming elemental compositions were typically within ±3 ppm accuracy. In all experiments a direct infusion technique was used, and samples prepared in a glovebox. Electron Paramagnetic Resonance (EPR) spectra were recorded on an X‐band Bruker Elexsys‐II E500 CW‐EPR spectrometer (Bruker Biospin GmbH, Rheinstetten, Germany) equipped with a high sensitivity cavity (SHQE1119) at 100±1 K. The instrument parameters were set as follows: microwave frequency, 9.43 GHz; modulation frequency, 100 kHz, and microwave power, 15.9 mW. The spectra were analyzed using Xepr software and the Anisotropic SpinFit simulation program (both Bruker Biospin GmbH).

### Syntheses

**[Co(NCN^CH2^−Et)Br] (1)**. To a solution of N(C−Br)N^CH2^−Et (215 mg, 0.65 mmol) in THF (10 mL) was slowly added *n*BuLi (0.45 mL, 1.6 M, 0.72 mmol) at −90 °C and then stirred at low temperature for 1 h. After allowing to warm to 0 °C, a suspension of anhydrous CoBr_2_ (150 mg, 0.68 mmol) in THF (5 mL) was added dropwise. The reaction mixture was stirred for further 30 min and all volatiles were removed under reduced pressure. The remaining dark solid was extracted into *n*‐pentane and the extract filtered through a syringe filter. The solvent was removed under reduced pressure to give a violet solid. Yield: 169 mg (67 %). μ_eff_=2.3(1) μ_B_. (benzene, Evans method). Elemental analysis: C_16_H_27_BrCoN_2_ (386.23) calc. C 49.75, H 7.05, N 7.25 found C 49.23, H 7.20, N 7.33.

**[Co(NCN^CH2^−Et)(NO)Br] (2)**. Nitric oxide was injected into the headspace of a solution of [Co(NCN^CH2^−Et)Br] (50 mg, 0.13 mmol) in THF (2 mL) whereupon the color changed from violet to dark purple. The reaction mixture was stirred for additional 15 min and all volatiles were removed under reduced pressure. Yield: 49 mg (91 %). ^1^H NMR (600 MHz, δ, C_6_D_6_): 7.01 (t, *J*=7.3 Hz, 1H, ph), 6.56 (d, *J*=7.6 Hz, 2H, ph), 3.26 (m, 2H, C*H*_2_NEt_2_), 2.96 (m, 4H, NC*H*_2_CH_3_), 2.76 (m, 2H, C*H*_2_NEt_2_), 2.49 (m, 4H, NC*H*_2_CH_3_), 0.61 (m, 4H, C*H*_3_), 0.54 (m, 8H, C*H*_3_). ^13^C{^1^H} NMR (151 MHz, δ, C_6_D_6_): 147.1 (s, ph*C*CH_2_), 124.8 (s, ph*C*H), 118.2 (s, ph*C*H), 60.7 (*C*H_2_NEt_2_), 52.8 (N*C*H_2_CH_3_), 51.9 (N*C*H_2_CH_3_), 10.6 (*C*H_3_), 9.0 (*C*H_3_). IR (ATR, cm^−1^): 1609 (ν_NO_). HR‐MS (ESI^+^, CH_3_CN) m/z calcd for C_16_H_27_CoN_3_O [M−Br]^+^ 336.1480 found 336.1483.

**EPR experiments. Reaction of 1 with L=pyridine,** ***t*** **BuNC and P(OMe)_3_. Formation of [Co(NCN^CH2^−Et)(L)Br] (3 a–c)**. To a solution of complex **1** (5 mg) in dry toluene (1 mL) was added an excess of ligand (pyridine, 30 μL; *t*BuNC, 5 mg; P(OMe)_3_, 30 μL) and the solution was stirred for 5 min. An aliquot was transferred to a heat‐dried EPR tube in a Glovebox and measured at 100 K revealing the formation of [Co(NCN^CH2^−Et)(L)Br]. EPR parameters: **3 a** g_1_=2.017. g_2_=2.228, g_3_=2.459; **3 b** g_1_=2.136, g_2_=2.110, g_3_=2.112 and **3 c** g_1_=2.073, g_2_=2.282, g_3_=2.076. (see Supporting Information for details)

**Reaction of 1 with CO. Formation of [Co(NCN^CH2^−Et)(CO)Br] (3 d)**. CO was injected into the headspace of a solution of **1** (10 mg) in toluene whereupon the color changed to green. All volatiles were removed and the solid was analyzed by IR spectroscopy. IR (ATR, cm^−1^): 1967 (ν_CO_).

**[Co(NCN^CH2^−Et)Br_2_] (4)**. To a solution of [Co(NCN^CH2^−Et)Br] (50 mg, 0.13 mmol) in THF (2 mL) was added solid CuBr_2_ (30 mg, 0.13 mmol) and the reaction mixture stirred for 5 min. After removal of the solvent under reduced pressure, CH_2_Cl_2_ (3 mL) was added, and the solution filtered through a syringe filter. All volatiles were removed and the obtained green solid washed with *n*‐pentane (5 mL). Yield: 53 mg (88 %). μ_eff_=4.8(1) μ_B_ (THF, Evans method). Elemental analysis: C_16_H_27_Br_2_CoN_2_ (466.14) calc. C 41.23, H 5.84, N 6.01 found C 41.66 H 5.97 N 5.91.

**Reaction of 1 with CH_3_CN and TlBF_4_ to form [Co(NCN^CH2^−Et)(MeCN)_3_](BF_4_)_2_ (5)**. To a solution of complex **4** (35 mg, 0.07 mmol) in dry acetonitrile (2 mL) was added TlBF_4_ (46 mg, 0.15 mmol). The reaction mixture was stirred for 3 h and all volatiles were removed under reduced pressure. The crude product was redissolved in CH_2_Cl_2_ (3 mL) and filtered through a syringe filter. The solvent was evaporated to afford a red‐brown solid. ^1^H NMR (400 MHz, δ, CD_3_CN): 7.76 (m, 1H, ph), 7.66 (m, 2H, ph), 4.13 (m, 4H, C*H*_2_NEt_2_), 3.04 (m, 8H, C*H*_2_CH_3_), 2.01 (bs, 9H, C*H*_3_CN), 1.42 (t, *J*=6.1 Hz, 12H, C*H*_3_).

### Computational Details

All calculations were performed using the ORCA 4.2.1 software package^[15]^ utilizing the Vienna Scientific Cluster (VSC3) in part. Electronic ground state calculations, including geometry optimizations and frequencies were carried out with density functional theory (DFT) using the GGA functional BP86^[16]^ and Ahlrichs^[17]^ def2‐TZVP basis set on all atoms. The resolution of identity (RI) approximation was used along with the corresponding auxiliary basis sets to accelerate the calculations. State averaged and state specific CAS‐SCF^[18]^ calculations were carried out with an active space comprised of seven electrons in five d‐orbitals using the obtained DFT‐geometry of the low spin species. To capture the effect of the dynamic correlation, NEVPT2 correction^[19]^ was employed on top of the CAS‐SCF wave function. The def2‐TZVP basis set and a very fine integration grid (Grid6) was used in all calculations. Orbital plots and graphics were generated with ChemCraft.^[20]^

### X‐Ray Structure Determination

X‐ray diffraction data of **1** and **2** (CCDC 2098276, 2098277) were collected at *T*=100 K in a dry stream of nitrogen on a Bruker Kappa APEX II diffractometer system using graphite‐monochromatized Mo‐*K*α radiation (λ=0.71073 Å) and fine sliced *ϕ*‐ and *ω*‐scans. Data were reduced to intensity values with SAINT and an absorption correction was applied with the multi‐scan approach implemented in SADABS.^[21]^ The structure was solved by the dual‐space approach implemented in SHELXT^[22]^ and refined against *F* ^2^ with SHELXL.^[23]^ Non‐hydrogen atoms were refined with anisotropic displacement parameters. The H atoms were placed in calculated positions and thereafter refined as riding on the parent C atoms. Molecular graphics were generated with the program MERCURY.^[24]^

Deposition Numbers 2098276 (for **1**) and 2098277 (for **2**) contain the supplementary crystallographic data for this paper. These data are provided free of charge by the joint Cambridge Crystallographic Data Centre and Fachinformationszentrum Karlsruhe Access Structures service www.ccdc.cam.ac.uk/structures.

## Conflict of interest

The authors declare no conflict of interest.

## Supporting information

As a service to our authors and readers, this journal provides supporting information supplied by the authors. Such materials are peer reviewed and may be re‐organized for online delivery, but are not copy‐edited or typeset. Technical support issues arising from supporting information (other than missing files) should be addressed to the authors.

Supporting Information
