# Supplementary material for: Synthesis and Characterization of Cobalt NCN Pincer Complexes
Source: Eur J Inorg Chem. 2021 Oct 11;2021(41):4280–5. doi: 10.1002/ejic.202100643 (PMC8596404; doi:10.1002/ejic.202100643)
Supplement: Supplementary file 1 — Supporting Information [file EJIC-2021-4280-s001.pdf]

European Journal of Inorganic Chemistry

Supporting Information

Synthesis and Characterization of Cobalt NCN Pincer Complexes

Jan Pecak, Wolfgang Eder, Gerald Tomsu, Berthold Stöger, Marc Pignitter, and Karl Kirchner*

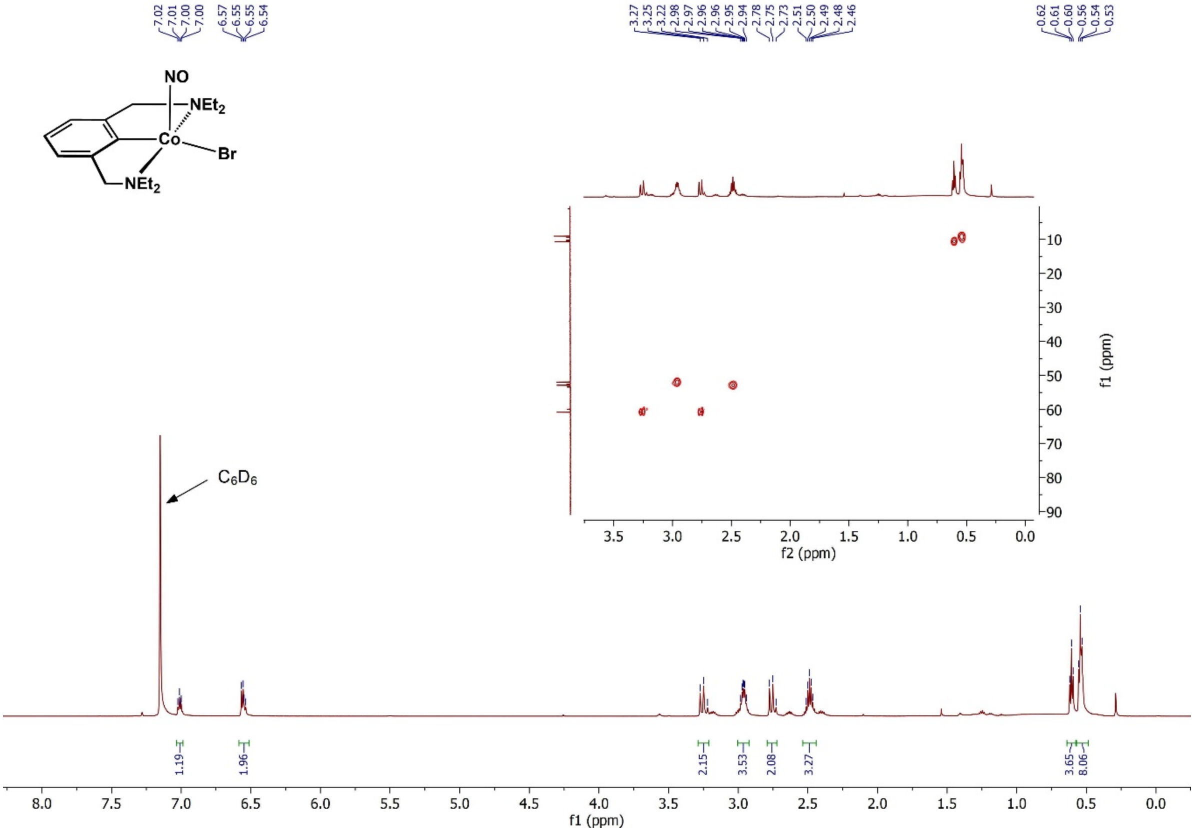

Fig. S1 ^{1}H NMR Spectrum of [Co(NCN^{CH2}-Et)(NO)Br] with ^{1}H,^{13}C-HSQC (insert).

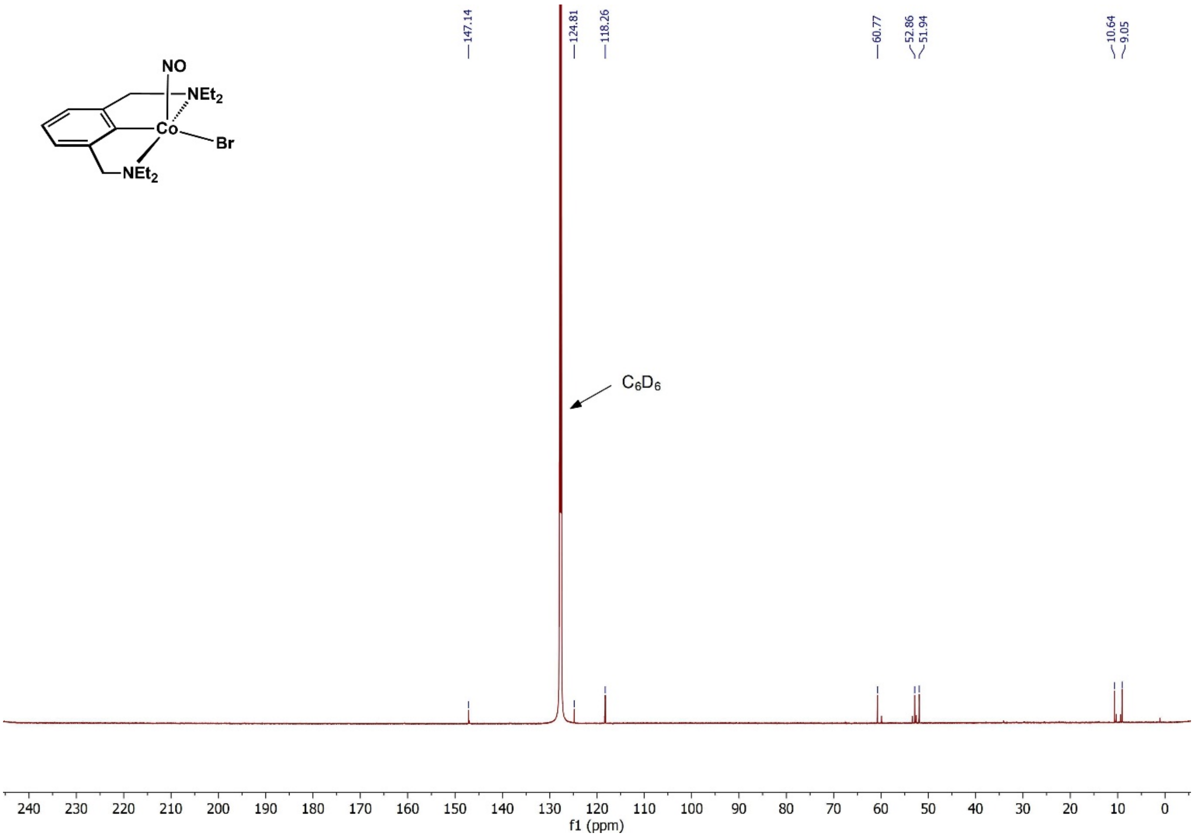

Fig. S2 $^{13}C\{^{1}H\}$ NMR Spectrum of [Co(NCN^{CH2}-Et)(NO)Br].

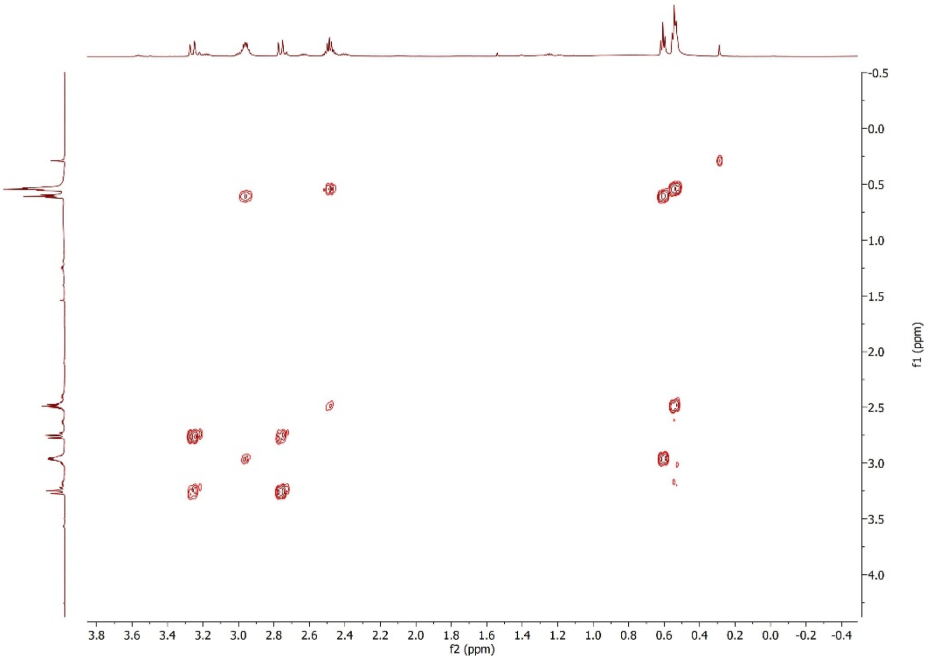

Fig. S3 ^{1}H,1H-COSY NMR Spectrum of [Co(NCN^{CH2}-Et)(NO)Br].

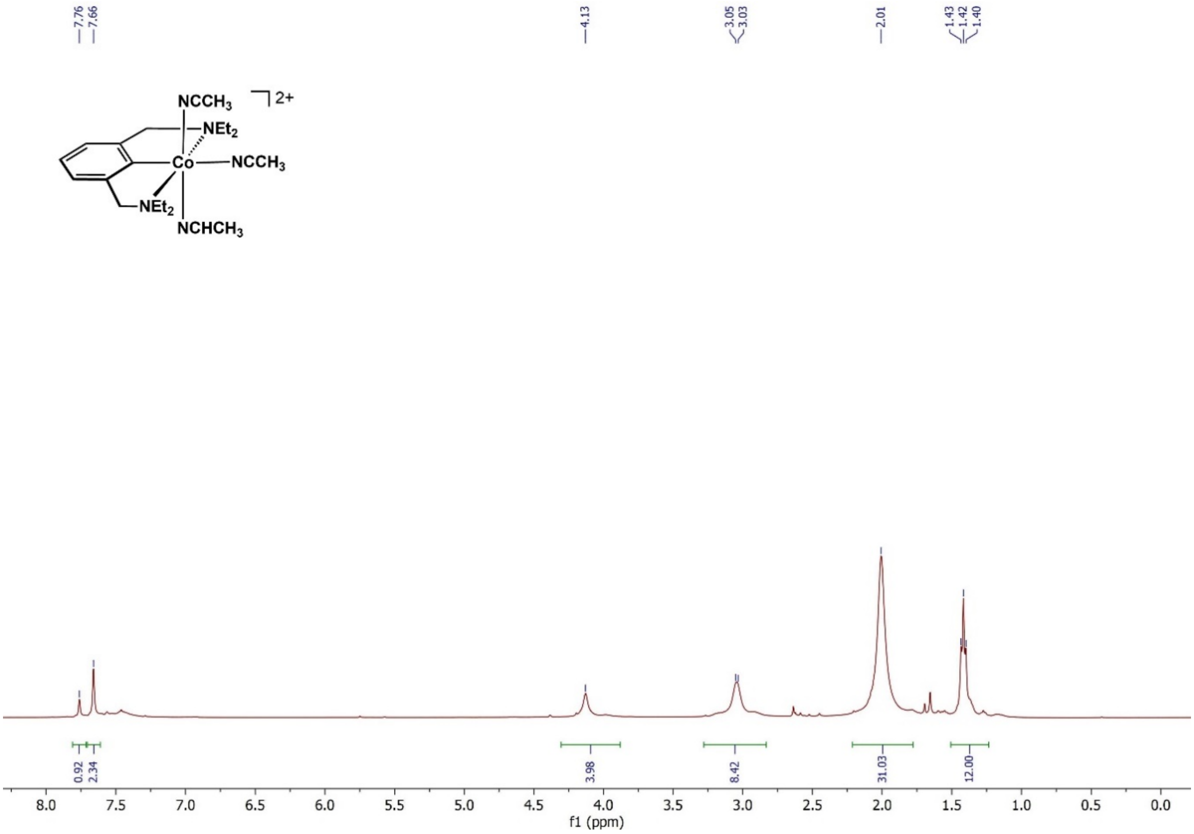

Fig. S4 ^{1}H NMR Spectrum of [Co(NCN^{CH2}-Et)(CH$_3$CN)$_3$](BF$_4$)$_2$.

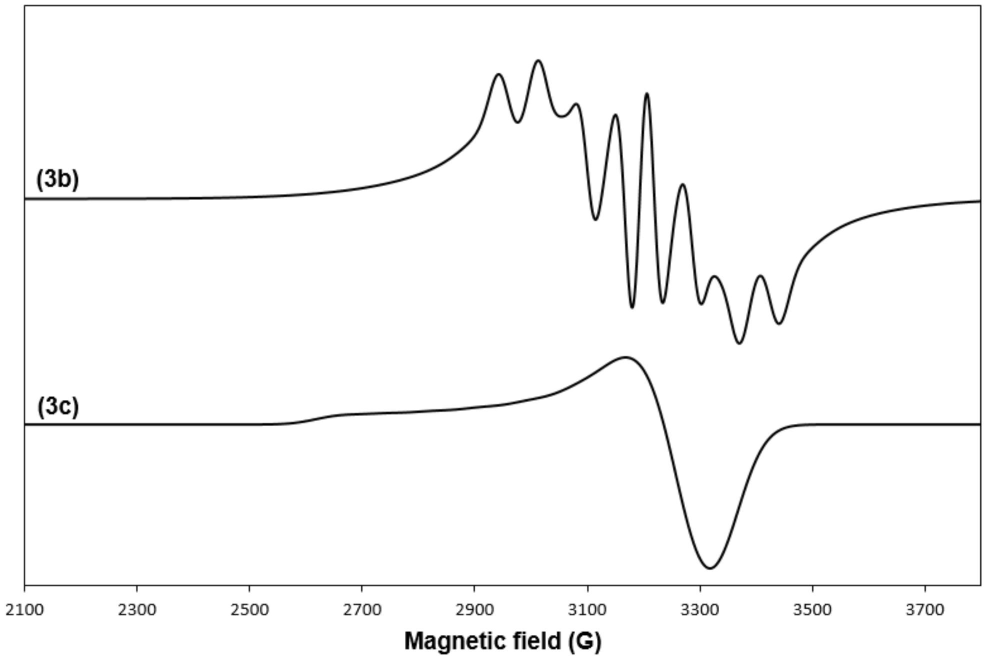

Fig. S6 X-Band EPR Spectra of **3b** and **3c** in toluene glass at 100 K.

Table S1 EPR parameters for all compounds (**1**, **3a-c**).

	g_1	g_2	g_3	A_1	A_2	A_3
[Co(NCN$^{\mathrm{CH2}}$-Et)Br] **(1)**	3.387	2.958	1.953	200	226	230
[Co(NCN$^{\mathrm{CH2}}$-Et)(py)Br] **(3a)**	2.017	2.228	2.459	58	*b*	9
[Co(NCN$^{\mathrm{CH2}}$-Et)(*t*BuNC)Br] **(3b)**	2.136	2.110	2.112	*b*	35	70
[Co(NCN$^{\mathrm{CH2}}$-Et)($C_3H_9O_3P$)Br] **(3c)**	2.073	2.282	2.076	7	83	*b*

[a] A is expressed in Gauss (G).
[b] Parameters not resolved properly.

Table S2 Specifications for the CAS-SCF/NEVPT2 calculation on **1** and **3a**.

```
%casscf   nel 7
          norb 5
          mult 2
          nroots 10
          trafostep ri
          nevpt2 sc
          rel
                  dosoc true
                  gtensor true
          end
end
```

```
ROOT   0:  E=   -4684.5668218588 Eh (1)
      0.95834 [29]: 22210
      0.00991 [23]: 22012
      0.00947 [22]: 21220
      0.00865 [15]: 20212
      0.00528 [2]:  02212
      0.00279 [24]: 22021
ROOT   0:  E=   -4931.3290633352 Eh (3a)
      0.95577 [    29]: 22210
      0.01679 [    23]: 22012
      0.01090 [    15]: 20212
      0.00664 [     2]: 02212
      0.00422 [     5]: 11122
```

Fig. S7 CAS-SCF Active space orbitals and energy (E_h) for complex **1**.

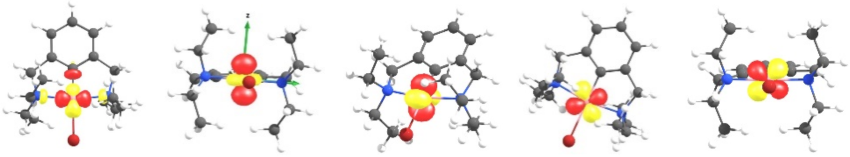

# 100	# 99	# 98	# 97	# 96
d_{x2-y2}	d_{z2}	d_{yz}	d_{xz}	d_{xy}
0.053	-0.312	-0.341	-0.395	-0.414

The active orbital set for molecule **3a** was chosen in a similar fashion comprising the 5 d-orbitals.

Table S3 Atomic Coordinates of complex **1** (DFT).

C	0.000000000	0.000000000	0.000000000
N	2.043431205	-1.630780068	0.000000000
Br	0.001533512	-4.298067224	0.003419125
Co	0.000000000	-1.844096108	0.000000000
N	-2.043949511	-1.635712356	-0.010171132
C	1.211207485	0.702511578	0.099303382
C	-1.220747067	2.098607023	-0.066631700
H	-2.159034606	2.656675735	-0.128651717

C	1.210956388	2.102492636	0.107927219
H	2.146334748	2.663925297	0.182969347
C	-0.006585481	2.793499999	0.029327002
H	-0.009552993	3.884557105	0.043188005
C	-1.214127931	0.699331421	-0.087516561
C	-2.417999348	-0.182347596	-0.256511714
H	-2.798178644	-0.105929761	-1.286626160
H	-3.259270428	0.091678000	0.404093747
C	-2.163277592	-2.388198149	-2.430221492
H	-1.139154753	-2.786712542	-2.450365368
H	-2.156817895	-1.358511764	-2.816600905
H	-2.769028503	-2.994199017	-3.119094014
C	-2.736714179	-2.499900803	-1.026458412
H	-3.813070267	-2.242801463	-1.016221994
H	-2.627082143	-3.536149615	-0.680854191
C	-2.542101133	-2.067188718	1.341318751
H	-2.313382863	-3.138656831	1.421086710
H	-3.642681273	-1.948859487	1.355259899
C	-1.920757967	-1.316464237	2.510426172
H	-0.831933644	-1.464584830	2.548558557
H	-2.344249656	-1.701891754	3.449314322
H	-2.109335459	-0.235751877	2.469732922
C	2.420283895	-0.176590785	0.245290838
H	2.820252519	-0.104473661	1.268165089
H	3.247620611	0.101698762	-0.430539443
C	2.739522825	-2.498325506	1.010797315
H	3.818404312	-2.252844183	0.985874169
H	2.613711321	-3.534041986	0.670275814
C	2.186051875	-2.375211686	2.421727223
H	2.794121136	-2.987454565	3.103359930
H	1.157313393	-2.760160136	2.458639541
H	2.198242316	-1.344582708	2.803734730
C	2.528958540	-2.064315110	-1.354950558
H	2.286696766	-3.133616376	-1.434160632
H	3.630930942	-1.959846609	-1.374553771
C	1.912455539	-1.304521987	-2.521119828
H	2.324910310	-1.699061179	-3.461194935
H	2.119305377	-0.226385506	-2.484776625
H	0.820708543	-1.432236566	-2.553983504

Table S4 Atomic Coordinates of complex **3a** (DFT).

Br	-0.265489208	2.422495910	-0.467075461
Co	0.000000000	0.000000000	0.000000000
N	-2.018721136	-0.502954706	-0.623898098
C	0.205263041	-1.843071903	-0.169817826
N	2.086600476	-0.049910968	-0.606452186
C	-0.933347343	-2.670235162	-0.302847485
C	-0.777341389	-4.052793834	-0.518249598
H	-1.659570113	-4.705929938	-0.628429005
N	0.000000000	0.000000000	2.055166160
C	0.517252974	-4.606090925	-0.603427167
H	0.639362873	-5.686493396	-0.775056286
C	1.656533182	-3.781449177	-0.495320416
H	2.662805658	-4.224123703	-0.587232354
C	1.499999831	-2.399320247	-0.280789345
C	-2.236633956	-1.921624044	-0.183191310
H	-2.557669780	-1.882359447	0.879018849
H	-3.072719941	-2.395683456	-0.747006496
C	-3.149777807	0.377739998	-0.203345334
H	-4.102709532	-0.070308291	-0.572204084
H	-3.001842133	1.340542782	-0.732314398
C	2.602653383	-1.380474171	-0.142401263
H	3.537855986	-1.665715241	-0.678514588
H	2.877613387	-1.261642572	0.926484794
C	-3.257877357	0.656175660	1.290817788
H	-4.184018901	1.235437113	1.482617054
H	-2.400468601	1.261755696	1.635288706
H	-3.312431199	-0.264304220	1.905364469

C	-1.865020676	-0.421998610	-2.119765208
H	-1.040051045	-1.111183308	-2.387493158
H	-1.518555020	0.611735485	-2.326631532
C	-3.093647843	-0.748362970	-2.980567753
H	-2.791885794	-0.713639051	-4.047173670
H	-3.917753869	-0.018541842	-2.854724103
H	-3.498982334	-1.764682171	-2.795703434
C	2.984942617	1.069601564	-0.193208675
H	2.590245419	1.974597110	-0.698161639
H	4.007070681	0.873219961	-0.593565643
C	3.073397145	1.344321827	1.302754178
H	3.412579878	0.467067279	1.889275750
H	2.098612726	1.679249813	1.698646982
H	3.808953570	2.155722530	1.475422735
C	3.216325703	-0.093306336	-2.950271039
H	3.822583833	-0.999669524	-2.743688092
H	3.867576999	0.794939533	-2.826439302
H	2.927453248	-0.136273273	-4.020236040
C	1.936053376	-0.023677113	-2.104776245
H	1.377467141	0.907726495	-2.330927470
H	1.280530723	-0.876448904	-2.368807778
C	-0.140626615	1.239490213	4.143248863
C	-0.122804278	1.170501726	2.745848998
H	-0.209308625	2.073019481	2.117456550
C	0.111568416	-1.137466993	2.795408797
C	0.102775208	-1.150477478	4.196107447
C	-0.026693736	0.058943027	4.897340902
H	0.211564532	-2.071684422	2.222094151
H	0.197898103	-2.111819367	4.723711632
H	-0.038663242	0.081254509	5.997673199
H	-0.245539284	2.221802909	4.628692638
